# Bacteria-derived DNA in serum extracellular vesicles are biomarkers for renal cell carcinoma

**DOI:** 10.1016/j.heliyon.2023.e19800

**Published:** 2023-09-06

**Authors:** Toshihiro Uemura, Atsunari Kawashima, Kentaro Jingushi, Daisuke Motooka, Takuro Saito, Sassi Nesrine, Toshiki Oka, Yohei Okuda, Akinaru Yamamoto, Gaku Yamamichi, Eisuke Tomiyama, Yu Ishizuya, Yoshiyuki Yamamoto, Taigo Kato, Koji Hatano, Kazutake Tsujikawa, Hisashi Wada, Norio Nonomura

**Affiliations:** a Department of Urology, Osaka University, Graduate School of Medicine, 2-2 Yamadaoka, Suita, Osaka, 565-0871, Japan; b Laboratory of Molecular and Cellular Physiology, Graduate School of Pharmaceutical Sciences, Osaka University, 1-6 Yamadaoka, Suita, Osaka, 565-0871, Japan; c Department of Infection Metagenomics, Genome Information Research Center, Osaka University Research Institute for Microbial Diseases, Suita, Osaka, 565-0871, Japan; d Department of Surgery, Graduate School of Medicine, Osaka University, Suita, Osaka, 565-0871, Japan; e Department of Clinical Research in Tumor Immunology, Graduate School of Medicine, Osaka University, Suita, Osaka, 565-0871, Japan

## Abstract

This is the first study to determine the clinical importance of circulating bacterial DNA in patients with renal cell carcinoma (RCC). We performed 16S rRNA metagenomic analysis of serum extracellular vesicles (EVs) from 88 patients with RCC and 10 healthy donors and identified three abundant bacterial DNA: Bacteroidia, TM7-1, and Sphingomonadales. Combining characteristic bacterial DNA information (three bacteria-derived DNA), a BTS index was created to diagnose patients with RCC. The BTS index showed high sensitivity not only in the discovery cohort, but also in the validation cohort, suggesting that it was useful as a screening test. Furthermore, in nivolumab treatment of RCC, patients with higher levels of Bacteroidia DNA in serum EVs had significantly poorer progression-free and overall survival than did those with lower levels. This study showed that circulating Bacteria-derived DNA could be used as a biomarker for RCC.

## Highlights

• To date, there is no clinically applied diagnostic biomarker for renal cell carcinoma.
• We established a BTS index for diagnosing renal cell carcinoma.
• This BTS index showed high sensitivity in the both discovery and validation cohorts.
• Thus, circulating Bacteria-derived DNA could be a biomarker for renal cell carcinoma.

Subject terms: Extracellular vesicles, Bacteria-derived DNA, Renal cell carcinoma.

## 1 Introduction

Early diagnosis of renal cell carcinoma (RCC) is desirable due to its poor prognosis in the advanced stages; However, there is no clinically applied diagnostic biomarkers [1,2]. Early stage RCC has few clinical symptoms and is often diagnosed incidentally during imaging tests such as echo and computed tomography (CT) [3,4]. To efficiently diagnose RCC, developing a simple and sensitive screening test is necessary.

Organ-specific bacterial flora has been reported to form in cancer tissues [5]. In addition, bacteria-derived DNA (b-DNA) is present in the blood of patients with cancer [6], demonstrating the presence of microorganisms in human tissues that were previously considered sterile. Bacterial extracellular vesicles (EVs) have been reported to be present in human blood and involved in signal transduction between bacteria and human host cells [7,8]. These findings suggest that bacteria influence various human diseases, including cancer.

PD-1-expressing regulatory T cells (Treg) in various tumor tissues have been reported to influence the efficacy of immune checkpoint inhibitor (ICI) therapy [9,10]. Tregs contribute to cancer progression by suppressing anti-tumor immunity [11]. We previously reported that CD4 + T cells infiltrating RCC tissues could be fractionated into five groups (Fr. I, II, III, IV, and V) according to PD-1 and TIM-3 expression intensity, and PD-1^low^TIM-3^+^ CD4 T cells (Fr. V) were characterized as Treg [12]. To predict the efficacy of ICI therapy in patients with RCC, it is important to evaluate factors associated with Tregs.

Extracellular vesicles are important mediators of communication between tumor and normal cells and are of interest as biomarkers [13,14]. We have previously reported that b-DNA in serum EVs could be useful for predicting the efficacy of ICI therapy in patients with urothelial carcinoma (UC) [15]. This suggests that bacterial information in the blood may influence the immune status of tumor tissues. To our best knowledge, this is the first study to assess the effectiveness of b-DNA included in serum EVs in diagnosing and predicting treatment response in patients with RCC.

## 2 Results

### 2.1 Collection of extracellular vesicles from serum and isolation of bacteria-derived DNA

**Fig. 1:**
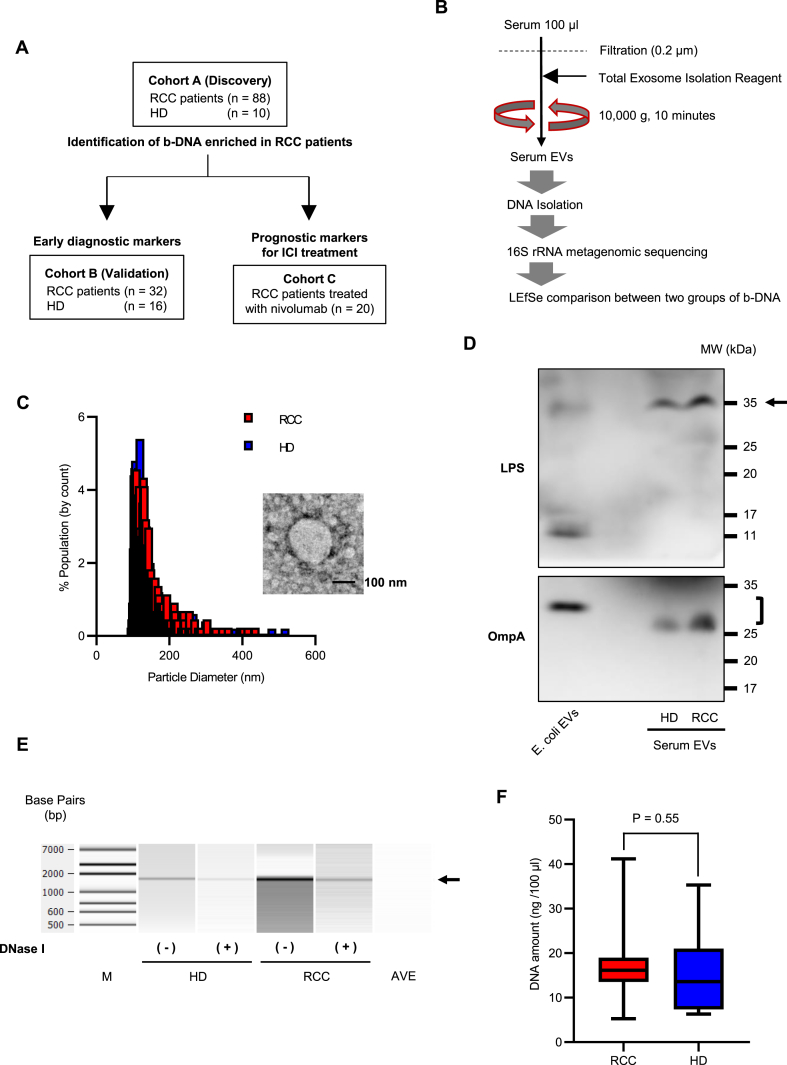
Extracellular vesicles (EVs) collection from serum and isolation of bacteria-derived DNA (b-DNA) (A) Study flow diagram. (B) Methods of processing and analyzing serum samples. (C) Representative results of nanoparticle and transmission electron microscopic analysis of EVs isolated from serum samples. A black bar indicates 100 nm. (D) Western blot analysis of serum EVs from a patient with renal cell carcinoma (RCC) and a healthy donor (HD) using an anti-E.coli LPS or an anti-OmpA antibody. (E) DNase I treatment of serum EVs followed by 16S rRNA gene amplification by PCR. Amplified PCR products are analyzed using Bioanalyzer. M: DNA ladder, AVE: buffer AVE. (F) DNA amount in EVs in serum 100 μL from 88 patients with RCC and 10 HD. Comparison between the two groups is performed by the Mann–Whitney *U* test.

A flow diagram of the study is shown in Fig. 1A. The clinical characteristics of 88 patients with RCC and 10 healthy donors (HD) are presented in Table 1 as a discovery Cohort A. There were no significant differences in age, body mass index (BMI), or sex between the two groups. The histology in all the patients revealed clear cell RCC. The pathological T classification of patients with RCC was T1 in 55 cases (62.5%), T2 in 5 cases (5.7%), and T3 in 28 cases (31.8%). Fifteen patients with RCC (17%) had a higher World Health Organization/International Society of Urological Pathology grade of RCC (Grade 3 or 4).

**Table 1:** Patient's characteristics of Cohort A.

Parameters		RCC (n = 88)	HD (n = 10)	*P*-value
Age at operation, years	Median (Range)	66 (38–82)	56 (26–73)	0.20
BMI, kg/m^2^	Median (Range)	22.6 (14.9–39.2)	23.9 (18.0–26.6)	0.73
Sex, n (%)	Male	63 (71.6)	6 (60.0)	0.48
	Female	25 (28.4)	4 (40.0)	
Histological type, n (%)	Clear cell RCC	88 (100)		
Pathological T stage, n (%)	T1	55 (62.5)		
	T2	5 (5.7)		
	T3	28 (31.8)		
Clinical N stage, n (%)	N0	87 (98.9)		
	N1	1 (1.1)		
Clinical M stage, n (%)	M0	77 (87.5)		
	M1	11 (12.5)		
Tumor grade, n (%)	1–2	73 (83.0)		
	3–4	15 (17.0)		

Abbreviations: BMI, body mass index; HD, healthy donor; RCC, renal cell carcinoma.

The same methods for collecting serum EVs and analyzing b-DNA were used for all cohorts (Fig. 1B). EVs isolated from serum samples were confirmed using nanoparticle analysis, transmission electron microscopy (TEM), and western blotting for lipopolysaccharide (LPS) or OmpA, a bacteria-derived EV marker (Fig. 1C and D). To determine presence of bacterial DNA in serum EVs, representative samples were treated with DNase I prior to DNA isolation. As shown in Fig. 1E, the bacterial DNA encoding the full-length 16S rRNA gene (approximately 1600 bp) was still detected after serum EVs were treated with DNase I, indicating that bacterial genomic DNA could be present inside circulating EVs. DNA was successfully isolated and detected from the collected serum EVs (median of DNA amount, RCC:16.2 ng/100 μl, HD:13.6 ng/100 μl) (Fig. 1F).

### 2.2 Differences in bacterial information based on various EV samples

**Fig. 2:**
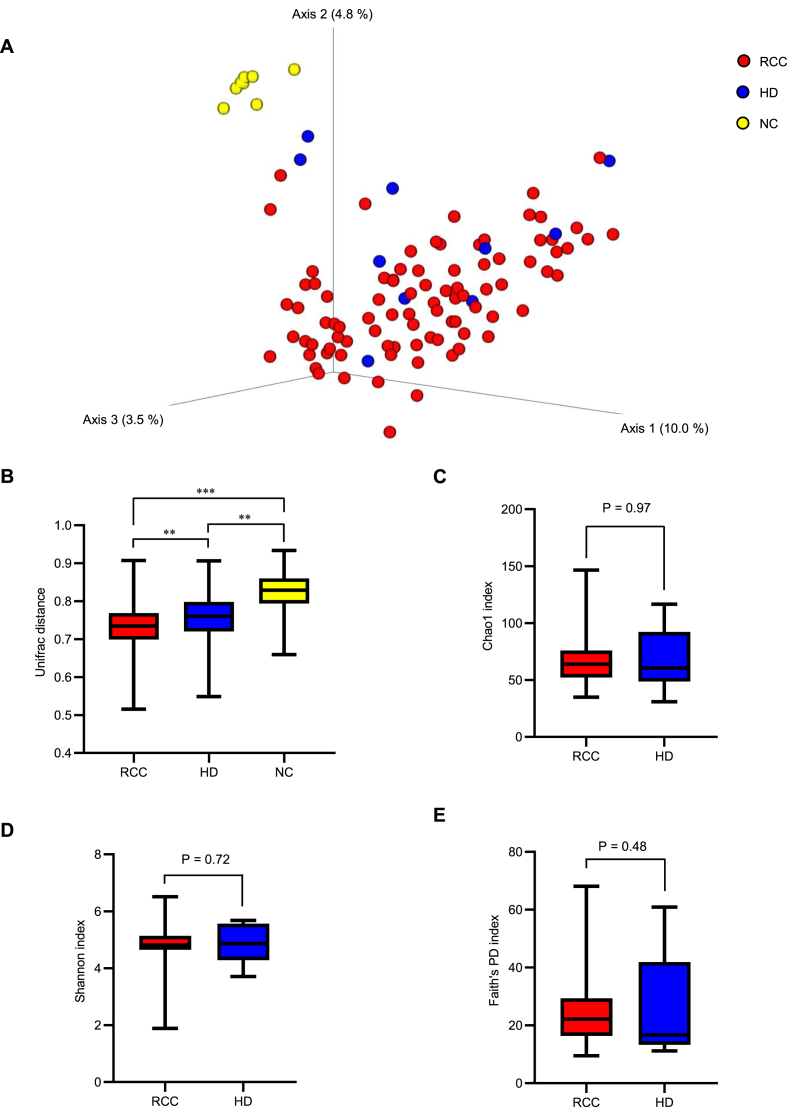
Differences in bacterial information from serum Extracellular vesicles (EVs) between patients with renal cell carcinoma (RCC) and healthy donors (HD) (A) Beta diversity plots for serum EVs from 88 patients with RCC, 10 HD, and negative control (NC). (B) Beta diversity analysis based on the uniFrac distance. A pairwise test from analysis of similarity (ANOSIM) is performed. (C–E) Alpha diversity analysis (C: Chao 1, D: Shannon, E: Faith's PD) for serum EVs from 88 patients with RCC and 10 HD. The Kruskal–Wallis test is performed. Significance values are *P < 0.05, **P < 0.01 and ***P < 0.001.

Polymerase chain reaction (PCR) was performed for metagenomic analyses using primers targeting the 16S ribosomal RNA (rRNA) gene (Table S1). In the principal coordinate analysis, b-DNA isolated from serum EVs in both HD and patients with RCC differed from that obtained from the negative control (NC: phosphate buffered saline) (Fig. 2A). The distance matrix revealed that b-DNA isolated from the serum EVs in patients with RCC significantly differed from that isolated from the serum EVs in HD (*P* < 0.01) and NC (*P* < 0.001) (Fig. 2B). There were no significant differences in the Chao1 index, Shannon index, or Faith's phylogenetic diversity (PD) index between HD and patients with RCC (Fig. 2C, D, E).

### 2.3 Identifying b-DNA enriched in serum EVs of patients with RCC

**Fig. 3:**
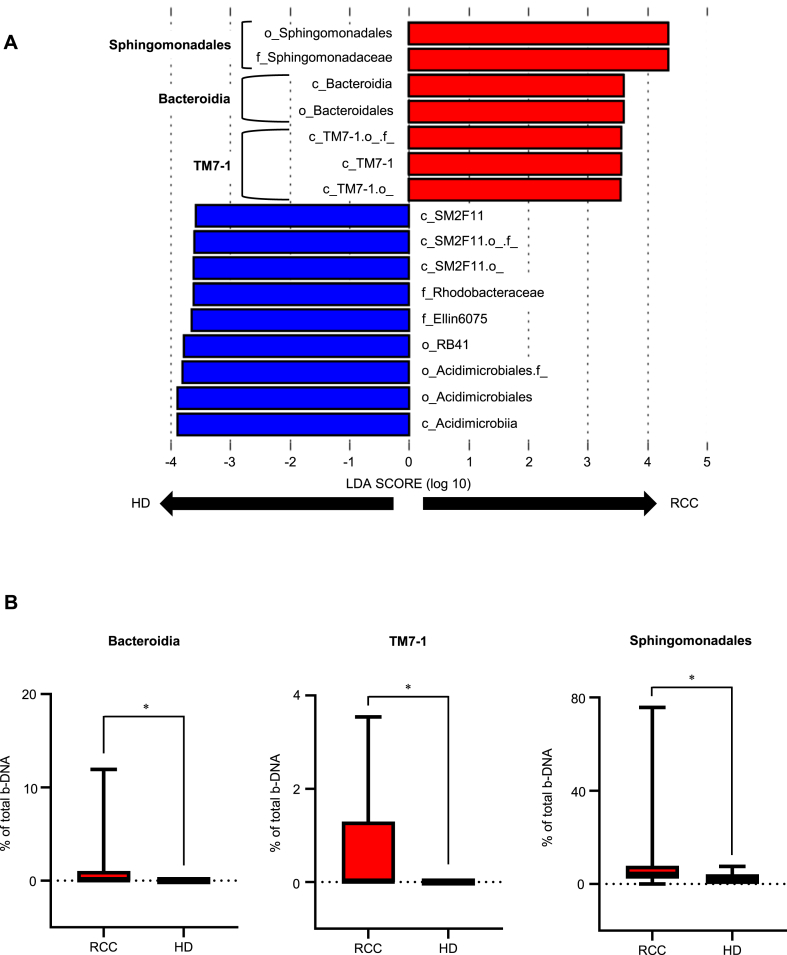
Comparison of the abundance of bacteria-derived DNA (b-DNA) in serum Extracellular vesicles (EVs) between patients with renal cell carcinoma (RCC) and healthy donors (HD) (A) Linear discriminant analysis Effect Size (LEfSe). Distinctive bacterial information detected in serum EVs from patients with RCC and HD. Biological classification classes are denoted by initials (c: class, f: family, o: order). (B) Three b-DNA abundant in serum EVs from patients with RCC. Mann–Whitney *U* test is performed. Significance values are *P < 0.05.

Linear discriminant analysis effect size (LEfSe) showed that seven operational taxonomic units (OTUs) had significantly higher relative abundance of the serum EVs in patients with RCC compared with the serum EVs in HD (Fig. 3A). At the class level, three groups (Bacteroidia, TM7-1, and Sphingomonadales) were identified as significantly having abundant serum EVs in patients with RCC: Bacteroidia (*P* < 0.05), TM7-1 (*P* < 0.05), and Sphingomonadales (*P* < 0.05) (Fig. 3B).

### 2.4 BTS index using b-DNA showed a high sensitivity for RCC in two different cohorts

**Fig. 4:**
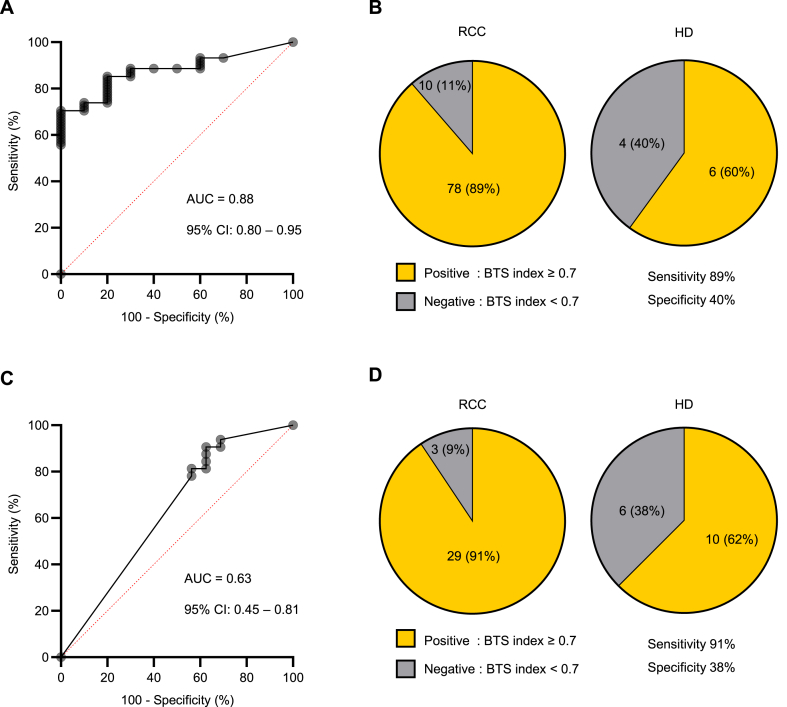
Discovery and validation of a novel biomarker using bacteria-derived DNAs (b-DNAs) to diagnose patients with renal cell carcinoma (RCC) (A) The ROC curve shows the diagnostic performance of the BTS index in patients with RCC in the discovery Cohort A. (B) Sensitivity and specificity with a BTS index value of 0.7 as a cut-off value in Cohort A. (C) The ROC curve shows the diagnostic performance of the BTS index in patients with RCC in the validation Cohort B. (D) Sensitivity and specificity with a BTS index value of 0.7 as a cut-off value in Cohort B. Sensitivity and specificity analysis are performed by Fisher's exact test.

The diagnostic performance of each identified b-DNA for RCC was 0.66 area under the curve (AUC) value, 33% sensitivity and 100% specificity for Bacteroidia, 0.68 AUC value, 35% sensitivity and 100% specificity for TM7-1 and 0.72 AUC value, 73% sensitivity and 70% specificity for Sphingomonadales. (Fig. S1A). Combining these three b-DNAs, we created a BTS index to diagnose RCC (BTS index = 1/(1 + Exp(-X)), X = 0.498482378 + Bacteroidia*259962.99858 + TM7-1 *64872.366873+ Sphingomonadales *20.9933271). The diagnostic performance of this index in patients with RCC had an AUC value of 0.88 (Fig. 4A). The cutoff value was set at 0.7 to achieve high sensitivity for the screening test, after which the sensitivity and specificity were 89% and 40%, respectively (Fig. 4B). Logistic regression analysis showed that the index was an independent factor for RCC diagnosis both in all patients (odds ratio [OR] 5.92, 95% confidence interval [CI]: 1.34–26.12, *P* = 0.02) (Table 2) and in patients with early-stage (pT1) RCC (OR 6.01, 95% CI 1.21–29.74, *P* = 0.03) (Table 3).

**Table 2:** Multiple logistic regression model of factors associated with RCC diagnosis in Cohort A.

Variable		Univariate			Multivariate	
		OR (95% CI)	P-Value		OR (95% CI)	*P*-value
Age	<65 Years	Reference			Reference	
	≥65 Years	1.97 (0.52–7.49)	0.32		2.19 (0.54–8.80)	0.27
Sex	Male	Reference			Reference	
	Female	0.60 (0.15–2.29)	0.45		0.48 (0.11–2.00)	0.31
BTS index	<0.7	Reference			Reference	
	≥0.7	5.20 (1.25–21.65)	**0.02**		5.92 (1.34–26.12)	**0.02**

Abbreviations: CI: confidence interval; OR: odds ratio.

Statistically significant P-values are shown in bold.

**Table 3:** Multiple logistic regression model of factors associated with RCC diagnosis in HD and patients with pT1 RCC in Cohort A.

Variable		Univariate			Multivariate	
		OR (95% CI)	P-Value		OR (95% CI)	*P*-value
Age	<65 Years	Reference			Reference	
	≥65 Years	1.56 (0.39–6.13)	0.53		1.54 (0.36–6.60)	0.56
Sex	Male	Reference			Reference	
	Female	0.56 (0.14–2.28)	0.42		0.42 (0.09–1.93)	0.26
BTS index	<0.7	Reference			Reference	
	≥0.7	5.44 (1.19–24.97)	**0.03**		6.01 (1.21–29.74)	**0.03**

The clinical characteristics of 32 patients with RCC and 16 HD are presented in Table 4, as a validation Cohort B for the BTS index. There were no significant differences in age, BMI, and sex between the two groups. The histology of all patients with RCC revealed the clear cell type. The pathological T classification in patients with RCC was T1 in 25 cases (78.1%) and T3 in 7 cases (21.9%). Six patients with RCC (18.7%) had a higher grade of RCC (grade 3 or 4). In Cohort B, the diagnostic performance of the BTS index in patients with RCC had an AUC value of 0.63, sensitivity of 91%, and specificity of 38% (Fig. 4C and D). Logistic regression analysis showed that the index was an independent factor for RCC diagnosis both in all patients (OR 5.95, 95% CI 1.23–28.77, *P* = 0.03) (Table 5) and in patients with pT1 RCC (OR 7.54, 95% CI 1.25–45.47, *P* = 0.03) (Table 6).

**Table 4:** Patient's characteristics of Cohort B.

Parameters		RCC (n = 32)	HD (n = 16)	*P*-value
Age at operation, years	Median (Range)	67 (51–88)	64 (26–75)	0.14
BMI, kg/m^2^	Median (Range)	24.4 (17.5–33.9)	23.7 (18.0–28.5)	0.26
Sex, n (%)	Male	23 (71.9)	10 (62.5)	0.53
	Female	9 (28.1)	6 (37.5)	
Histological type, n (%)	Clear cell RCC	32 (100)		
Pathological T stage, n (%)	T1	25 (78.1)		
	T2	0		
	T3	7 (21.9)		
Clinical N stage, n (%)	N0	32 (100)		
	N1	0		
Clinical M stage, n (%)	M0	31 (96.9)		
	M1	1 (3.1)		
Tumor grade, n (%)	1–2	26 (81.3)		
	3–4	6 (18.7)

**Table 5:** Multiple logistic regression model of factors associated with RCC diagnosis in Cohort B.

Variable		Univariate			Multivariate	
		OR (95% CI)	*P*-Value		OR (95% CI)	*P*-value
Age	<65 Years	Reference			Reference	
	≥65 Years	1.13 (0.34–3.77)	0.84		1.43 (0.37–5.52)	0.61
Sex	Male	Reference			Reference	
	Female	0.65 (0.18–2.33)	0.51		0.57 (0.14–2.37)	0.44
BTS index	<0.7	Reference			Reference	
	≥0.7	5.80 (1.22–27.63)	**0.03**		5.95 (1.23–28.77)	**0.03**

**Table 6:** Multiple logistic regression model of factors associated with RCC diagnosis in HD and patients with pT1 RCC in Cohort B.

Variable		Univariate			Multivariate	
		OR (95% CI)	*P*-Value		OR (95% CI)	*P*-value
Age	<65 Years	Reference			Reference	
	≥65 Years	1.08 (0.31–3.80)	0.90		1.25 (0.31–5.10)	0.75
Sex	Male	Reference			Reference	
	Female	0.64 (0.17–2.47)	0.52		0.50 (0.11–2.22)	0.36
BTS index	<0.7	Reference			Reference	
	≥0.7	6.90 (1.18–40.27)	**0.03**		7.54 (1.25–45.47)	**0.03**

We examined whether the BTS index could distinguish between HD and patients with bladder cancer and found that the AUC value was 0.52, suggesting that it would not be useful, except for RCC (Fig. S1B, Table S2).

### 2.5 Relative abundance of bacteroidia DNA in serum EVs correlated with intratumoral treg in RCC tissues

**Fig. 5:**
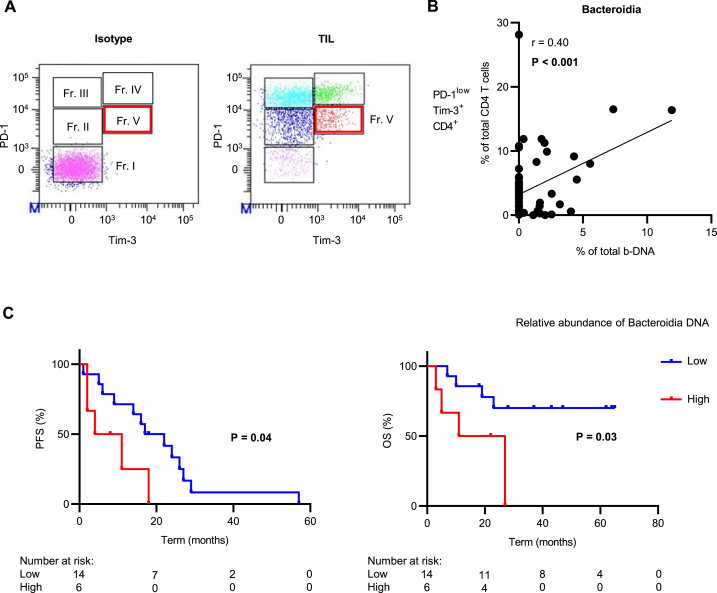
Correlation between bacteria-derived DNA (b-DNA) and tumor-infiltrating lymphocytes (TILs) (A) Co-expression pattern of PD-1 and TIM-3 on CD4 T cells of TILs in 88 patients with RCC in Cohort A. CD4 T cells are fractionated into 5 groups (Fr. I: PD-1^−^TIM-3^-^, Fr. II: PD-1^low^TIM-3^-^, Fr. III: PD-1^high^TIM-3^-^, Fr. IV: PD-1^high^TIM-3^+^, Fr. V: PD-1^low^TIM-3^+^). (B) Correlation between the relative abundance of Bacteroidia DNA in serum EVs and PD-1^low^TIM-3^+^CD4 T cells in RCC tissue. (C) Overall survival (OS) and progression-free survival (PFS) after initiation of nivolumab monotherapy for advanced RCC by Bacteroidia DNA in serum EVs. Statistical analysis was performed by log-rank test.

Serum and paired RCC tissue samples from 88 patients with RCC in Cohort A were used for comparing blood bacterial information and intratumoral immune status. Representative expression patterns of PD-1 and TIM-3 in the CD4 + T cells of tumor-infiltrating lymphocytes (TILs) are shown in Fig. 5A. As in our previous report [12], CD4 T cells were fractionated into five groups (Fr. I: PD-1^−^TIM-3^-^, Fr. II: PD-1^low^TIM-3^-^, Fr. III: PD-1^high^TIM-3^-^, Fr. IV: PD-1^high^TIM-3^+^, Fr. V: PD-1^low^TIM-3^+^). Of these, Fr. V represents the CD4 + Treg-enriched cell population.

In Cohort A, we observed a significant positive correlation between CD4 PD-1^low^TIM-3^+^ T cells (Fr. V) and relative abundance of Bacteroidia DNA in serum EVs (r = 0.40, 95% CI: 0.19–0.57, *P* < 0.01) (Fig. 5B) (Table S3). The 75th percentile of relative abundance of Bacteroidia DNA in 88 patients with RCC in Cohort A was 1%. High Bacteroidia was defined as a relative abundance of Bacteroidia DNA ≥1% and low Bacteroidia as a relative abundance of Bacteroidia DNA <1%. There were no significant differences in progression-free survival (PFS) or overall survival (OS) after surgery based on a relative abundance of Bacteroidia DNA (Fig. S2).

The clinical characteristics of 20 patients with advanced RCC who received nivolumab monotherapy are shown in Table 7. The patients were divided into high or low Bacteroidia groups. There were no significant differences in age, Karnofsky Performance Status (KPS), sex, number of treatment lines, or presence of anemia between the two groups. A significant difference was observed only in tumor grade. The high Bacteroidia group had significantly poorer progression-free and overall survival than did the low Bacteroidia group (median PFS:8 months versus 20 months, *P* = 0.04) (median OS:19 months versus not reached, *P* = 0.03) (Fig. 5C).

**Table 7:** Patient's characteristics of Cohort C.

Parameters		Low Bacteroidia (n = 14)	High Bacteroidia (n = 6)	*P*-value
Age at operation, years	Median (Range)	66 (45–81)	62 (53–68)	0.14
KPS, n (%)	90–100	10 (71.4)	3 (50.0)	0.36
	70–80	4 (28.6)	3 (50.0)	
Sex, n (%)	Male	12 (85.7)	5 (83.3)	0.89
	Female	2 (14.3)	1 (16.7)	
Histological type, n (%)	Clear cell RCC	14 (100)	6 (100)	
Treatment line, n (%)	2nd	8 (57.1)	4 (66.7)	0.78
	3rd	5 (35.7)	2 (33.3)	
	4th	1 (7.2)	0	
Previous other ICI treatment, n (%)	Absence	14 (100)	6 (100)	
	Presence	0	0	
Anemia, n (%)	Absence	5 (35.7)	2 (33.3)	0.92
	Presence	9 (64.3)	4 (66.7)	
Tumor grade, n (%)	1–2	8 (57.1)	0	**0.02**
	3–4	5 (35.7)	3 (50.0)	
	unknown	1 (7.2)	3 (50.0)	

## 3 Discussion

EVs are universal intercellular signaling mechanisms common to the three domains of eukaryotes, bacteria, and archaea [16]. Reports suggest that EVs containing b-DNA in the human systemic circulatory system could spread to other organs and tissues [7,17]. In this study, we found that b-DNA in serum EVs was useful for early diagnosis and prediction of the efficacy of immunotherapy in patients with RCC.

In studies using low-biomass samples such as blood, contamination of DNA in DNA extraction kits and other laboratory reagents could affect the results [18]. Principal coordinate analysis and distance matrices revealed that the serum samples contained different bacterial information than did the negative control samples (Fig. 2A and B), suggesting that the effect of contamination was minimal.

Systemic evaluation using imaging is essential for treating RCC. Therefore, the most important requirements for diagnostic biomarkers for screening purposes are simplicity and high sensitivity. Intestinal bacterial composition has been reported to differ between patients and healthy controls [19,20]. In addition, the composition of bacterial population in RCC tissue could differ from that in normal renal tissue [21,22]. Based on these findings, b-DNA in serum EVs is expected to differ between patients with RCC and healthy controls, which is consistent with our study results. The BTS index had an AUC value of 0.63 in the validation cohort, which was not sufficient, but its sensitivity was high (89% and 91%) in two different cohorts (Fig. 4B, D), suggesting that it is useful as a screening test. Cell-free DNA and circulating tumor DNA have been reported as promising blood biomarker candidates for RCC [23,24]. The advantage of circulating b-DNA is that it is useful even for early-stage RCC with a low tumor volume (Table 3, Table 6).

Tumor-associated microbiota is an essential component of the tumor microenvironment of various types of human cancers [5,25]. It has been reported that the bacterial flora in tumor tissues could influence the intratumoral immune status [26] and Bacteroidia could increase Treg infiltration [27]. We previously reported that a population of PD-1^low^TIM-3^high^ CD4 T cells (Fr. V) increased the FOXP3 expression, which is specific to Tregs [12]. Our study results showed a positive correlation between Tregs in RCC tissues and bacterial information in the blood (Fig. 5B). Similarly, in patients with UC, blood bacterial information could reflect the intratumoral immune status [11], suggesting that blood bacterial information could be used as a biomarker for predicting the efficacy of ICI therapy.

In cohort C, more specimens with high Bacteroidia have high tumor grade, which raises the question whether the difference in tumor grade may have resulted in poor results (Table 7, Fig. 5C). Due to the small sample size, performing multivariate analysis in cohort C was difficult. In Cohort A, we found no correlation between Bacteroidia DNA in blood and tumor grade (Fig. S3). Therefore, although tumor grade was a confounding factor, it is not considered problematic since the purpose of this study was to demonstrate usefulness of b-DNA as a blood biomarker.

There are several reports on bacterial DNA in serum EVs in patients with non-urologic cancers. Kim et al. noted a predominance of Acinetobacter in the ovarian cancer group compared with the benign ovarian tumor group [28]. An et al. also noted that Gammaproteobacteria, Clostridia, Bacteroidia, Negativicutes, and Coriobacteriia were more abundant at the class level in the breast cancer group compared the control group [29]. In this study, Bacteroidia DNA in blood and Treg in tumor tissuesshowed a positive correlation. In addition, Bacteroidia could be higher in cancer types in which Treg was strongly involved.

### 3.1 Limitations of the study

First, the study was based only on Japanese data. Gut and urinary microbiota have been reported to vary according to genetic or environmental factors [30], and b-DNA in serum EVs is expected to be affected by race, diet, and other factors. Second, only 16S rRNA metagenomic analysis was used for identifying b-DNA in serum EVs. The shotgun metagenomic analysis is recommended for identifying bacterial species and the analysis of gene function [31,32]. However, the amount of b-DNA in serum EVs was low. Therefore, detection by shotgun metagenomic analysis without PCR could be difficult. It is expected that technical and cost issues will be resolved in the future and that research on b-DNA in EV using shotgun metagenomics will progress. Third, this was a retrospective analysis with a small sample size. In the discovery cohort, we could not collect a sufficient sample size of HD. In addition, although only nivolumab monotherapy was considered in this study, treatment combining multiple ICIs or combining ICI with a tyrosine kinase inhibitor should also have been considered [33,34]. However, immunotherapy for RCC is changing rapidly, and it was difficult to collect a large number of cases treated with the same regimen.

## Author contribution statement

Toshihiro Uemura; Atsunari Kawashima; Kentaro Jingushi: Conceived and designed the experiments; Performed the experiments; Analyzed and interpreted the data; Contributed reagents, materials, analysis tools or data; Wrote the paper.

Daisuke Motooka: Analyzed and interpreted the data; Contributed reagents, materials, analysis tools or data.

Takuro Saito; Sassi Nesrine; Toshiki Oka; Yohei Okuda; Akinaru Yamamoto; Gaku Yamamichi; Eisuke Tomiyama; Yu Ishizuya; Yoshiyuki Yamamoto; Taigo Kato; Koji Hatano; Kazutake Tsujikawa; Hisashi Wada; Norio Nonomura: Analyzed and interpreted the data; Contributed reagents, materials, analysis tools or data; Wrote the paper.

## 4 Data availability statement

Data will be made available on request.

**Ethics statement:** Clinical specimens were collected at the Osaka University Hospital (Osaka, Japan). The study was approved by the Ethics Review Board of Osaka University Medical Hospital (#13397-2, #14069-3) and was conducted in accordance with the Declaration of Helsinki. Written informed consent was obtained from all patients.

**Declaration of interests:** The authors declare no competing interests.

## 5 Star methods

### 5.1 Key resources table

The reagents/resources used in this study are listed in Table 8. The source of purchase and catalog identifier are also listed.

**Table 8:** Key resources table.

REAGENT or RESOURCE	SOURCE	IDENTIFIER
Antibodies		
Anti-E.coli LPS antibody	Abcam	Cat# ab35654
Anti E.coli OmpA Polyclonal Antibody	Antibody research	Cat# 111120
Anti-mouse IgG, HRP-linked	Cell Signaling	Cat# 7076
BV711 Anti-Human CD4	BioLegend	Cat# 317439, RRID: AB_11219404
PE-Cy7 Mouse anti-human CD279 (PD-1)	BD Biosciences	Cat# 561272, RRID: AB_10611585
APC anti-human CD366 (Tim-3)	BioLegend	Cat# 345011, RRID: AB_2561717
Chemicals, peptides, and recombinant proteins		
ECL detection reagent	GE Healthcare	Cat# RPN2235
DNase I	Nippon Gene	Cat# 314-08071
KAPA HiFi HS ReadyMix	Kapa Biosystems	Cat# KK2602
AMPure XP	Beckman Coulter	Cat# A63880
Human TruStain FcX	BioLegend	Cat# 422302
Critical commercial assays		
Total Exosome Isolation Reagent (from serum)	Thermo Fisher	Cat# 4478360
QIAamp® Circulating Nucleic Acid Kit	Qiagen	Cat# 55114
Qubit ® dsDNA HS Assay Kits	Thermo Fisher	Cat# Q32851
Bioanalyzer High Sensitivity DNA Kit	Agilent	Cat# 5067-4626
Zombie NIR™ Fixable Viability Kit	Biolegend	Cat# 423105
Software and algorithms		
QIIME	Illumina	http://qiime.org/1.3.0/index.html#
Galaxy	The Huttenhower Lab	http://huttenhower.sph.harvard.edu/galaxy/
Izon Control Suite	Izon	n/a
BD FACSDiva	BD Biosciences	n/a
JMP	SAS Institute	V16.1
Prism	GraphPad	V9.2
Other		
qNano Gold	Izon	n/a
HT7800	HITACHI	n/a
Amersham Imager 680	GE Healthcare	n/a
LSRFortessa X20 flow cytometer	BD Biosciences	n/a

### 5.2 Resource availability

**Lead contact**.

Further information and requests for resources and reagents should be directed to and will be fulfilled by the lead contact, Atsunari Kawashima (kawashima@uro.med.osaka-u.ac.jp).

**Materials availability**.

This study did not generate new unique regents.

**Data and code availability:** Data generated in this study are available from the corresponding author upon request.

## 6 Experimental model and subject details

### 6.1 Patient cohort

To develop novel diagnostic biomarkers, 88 patients with clear cell RCC who underwent radical or partial nephrectomy and 10 HD were enrolled in a discovery Cohort A. Thirty-two patients with clear cell RCC and 16 HD were enrolled in a validation Cohort B. To examine the diagnostic performance in other cancer types, 50 patients with bladder cancer who underwent transurethral resection of bladder tumors and 20 HD were enrolled in Cohort Z. To investigate predictive biomarkers for treatment response, a total of 20 patients treated with nivolumab monotherapy for advanced clear cell RCC were enrolled in Cohort C. There were no duplications of HD in any cohort.

### 6.2 Biomarker discovery

EVs were collected from the serum just before surgery and prior to antibiotic administration. Furthermore, metagenomic sequencing was performed after amplification of the 16S ribosomal RNA gene. In Cohort A, significantly abundant b-DNA in the serum EVs of patients with RCC was identified. These b-DNAs were combined to create a useful index for diagnosing patients with RCC. The diagnostic performance of the established index in patients with RCC was validated using Cohort B.

## 7 Method details

### 7.1 Sample preparation

Whole blood (2.0–7.0 mL) samples were collected directly into Venoject II tubes (TERUMO, Tokyo, Japan). Within 3 h of collection, all samples were centrifuged at 1200×*g* for 15 min, and supernatants, obtained as serum, were stored at −80 °C. Serum samples were centrifuged at 2000×*g* for 30 min and filtered with a 0.2 μm syringe filter (KURABO, Osaka, Japan) before being subjected to EV isolation. Serum EVs were isolated using Exosome Isolation Kit (serum) (Thermo Fisher Scientific, Waltham, MA, USA) according to the manufacturer's protocol.

### 7.2 Nanoparticle measurement

The size and concentration of the EVs were determined using qNano Gold (Izon Science, Christchurch, New Zealand). Data were analyzed using Izon Control Suite Software (V3.February 3, 2001).

### 7.3 Transmission electron microscopy analysis

The TEM analysis was performed according to the method described by Cecilia et al. [35]. EV samples were placed on a Formvar carbon-coated nickel grid for 1 h and fixed with 2% paraformaldehyde before observation with an HT7800 (HITACHI, Tokyo, Japan).

### 7.4 Western blot analysis

EV samples were lysed with Laemmli SDS sample buffer without 2-mercaptoethanol and separated on a 12% gel by SDS-polyacrylamide gel electrophoresis (PAGE), followed by transfer onto a polyvinylidene difluoride (PVDF) membrane using a Bio-Rad semidry transfer system (1 h, 12 V). Membranes were probed with anti-E.coli LPS (1:1000) or anti-OmpA (1:1000) primary antibodies. The membranes were then incubated with horseradish peroxidase (HRP)-conjugated secondary antibody against mouse immunoglobulin (1:5000, Cell Signaling Technology), followed by detection using enhanced chemiluminescence (ECL) western blotting detection reagent (GE Healthcare, Chicago, IL, USA). Chemiluminescence was detected using an Amersham Imager 680 (GE Healthcare). Since OmpA varies in length among bacterial species, the serum EV protein was detected at different locations than in E. coli [36].

### 7.5 DNase I treatment of serum EVs

EV samples were suspended in 100 μl of PBS and divided equally into two samples. One sample was treated with 0.24 U/μl DNase I (Nippon Gene) at 37 °C for 45 min to digest cell-free DNA and external DNA associated with EVs.

### 7.6 DNA isolation

DNA from serum EVs samples was isolated using the QIAamp® Circulating Nucleic Acid Kit (QIAGEN, Hilden, Germany) according to the manufacturer's protocol and measured using Qubit ® dsDNA HS Assay Kits (Thermo Fisher Scientific).

### 7.7 PCR amplification of 16S rRNA gene

The primer sets used for 16S rRNA PCR amplification are listed in Table S1. For the V1–V2 region: one PCR cycle consisted of a denaturation step at 98 °C for 20 s, annealing at 60 °C for 15 s, and extension at 72 °C for 15 s till 25 cycles. The full-length 16S rRNA gene was PCR-amplified using the primers 27F (5′-AGAGTTTGATCMTGGCTCAG-3′) and 1492R (5′-CGGTTACCTTGTTACGACTT-3′). For the full-length 16S rRNA gene: a PCR run of 25 cycles was carried out with denaturation at 95 °C for 30 s, annealing at 60 °C for 30 s, and extension at 72 °C for 90 s. Amplicons were analyzed using a Bioanalyzer High Sensitivity DNA Kit (Agilent, Santa Clara, CA, USA).

### 7.8 16S metagenomic sequencing

The PCR-amplified V1–V2 regions of the bacterial 16S rRNA gene (as mentioned above for PCR amplification of the 16S rRNA gene) were sequenced on a MiSeq platform (Illumina, San Diego, CA). QIIME version 2.202002 was used to process the raw sequencing data. The Chao1 index indicates species richness. The Shannon index indicates species richness and evenness. The Faith's PD index is a measure of biodiversity using the total length of the phylogenetic tree branches. Linear discriminant analysis Effect Size [37] was used to compare b-DNA between the RCC and HD groups. LEfSe was calculated using the Galaxy web platform, and only linear discriminant analysis (LDA) scores ≥3.5 were listed.

### 7.9 Multicolor flow cytometry

TILs were extracted macroscopically from cancer tissues immediately after surgery, and all fresh samples were analyzed using flow cytometry. In several cases where coagulative necrosis was difficult to distinguish macroscopically, the extracted area was evaluated using hematoxylin and eosin staining. Details of the lymphocyte extraction, methods, and antibody clones for staining cell surface molecules have been previously reported [38].

### 7.10 Statistics

Statistical analyses and visual quantification were performed using JMP software (JMP 16.1, SAS Institute) and GraphPad Prism software (GraphPad Prism 9.2, GraphPad software). Differences between values were statistically analyzed using the Mann-Whitney *U* test for two groups and the Kruskal–Wallis test for three groups. Diagnostic abilities were evaluated using ROC characteristics and logistic regression analyses. The formula of BTS index was created using logistic regression analysis. The sigmoid function "y = 1/(1+e^-x^) = 1/(1+exp(-x))" is used for the output of logistic regression model. The Kaplan–Meier method was used for calculating survival rates, and log-rank tests were used for comparing between the two groups. Statistical significance was set at *P* < 0.05.

## Declaration of competing interest

The authors declare that they have no known competing financial interests or personal relationships that could have appeared to influence the work reported in this paper.
