# Supplementary figures and images for: Bacteria-derived DNA in serum extracellular vesicles are biomarkers for renal cell carcinoma
Source: Heliyon. 2023 Sep 6;9(9):e19800. doi: 10.1016/j.heliyon.2023.e19800 (PMC10559165; doi:10.1016/j.heliyon.2023.e19800)

Figure S1

A

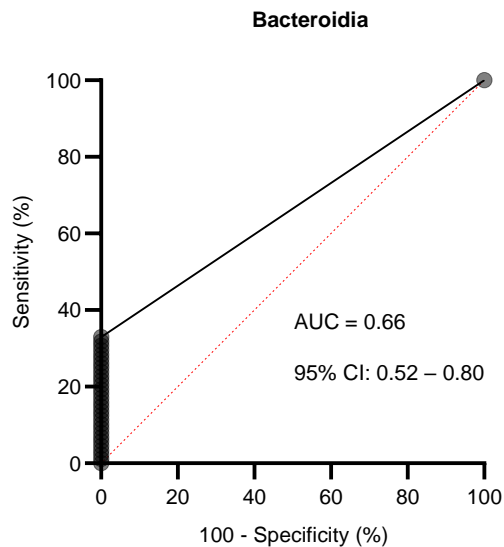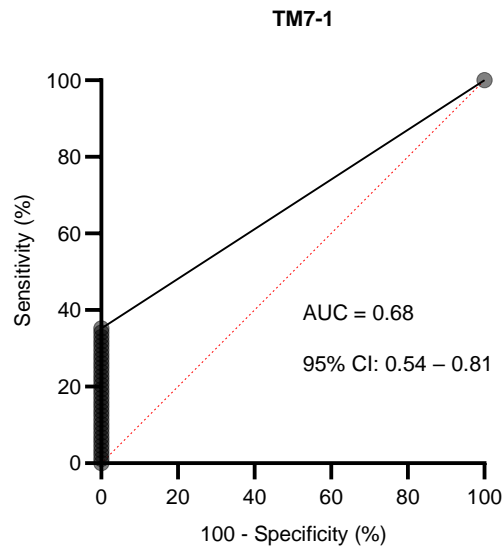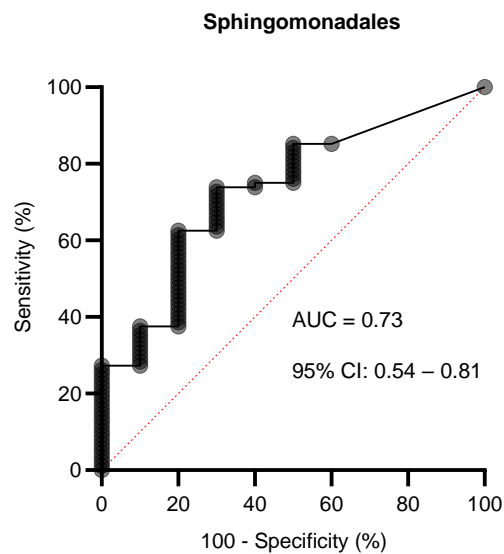

Supplement: Multimedia component 1 [file mmc1.pdf]

Figure S1

B

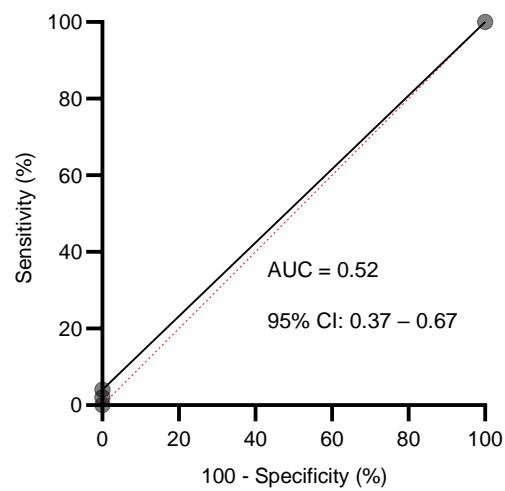

Supplement: Multimedia component 2 [file mmc2.pdf]

Figure S2

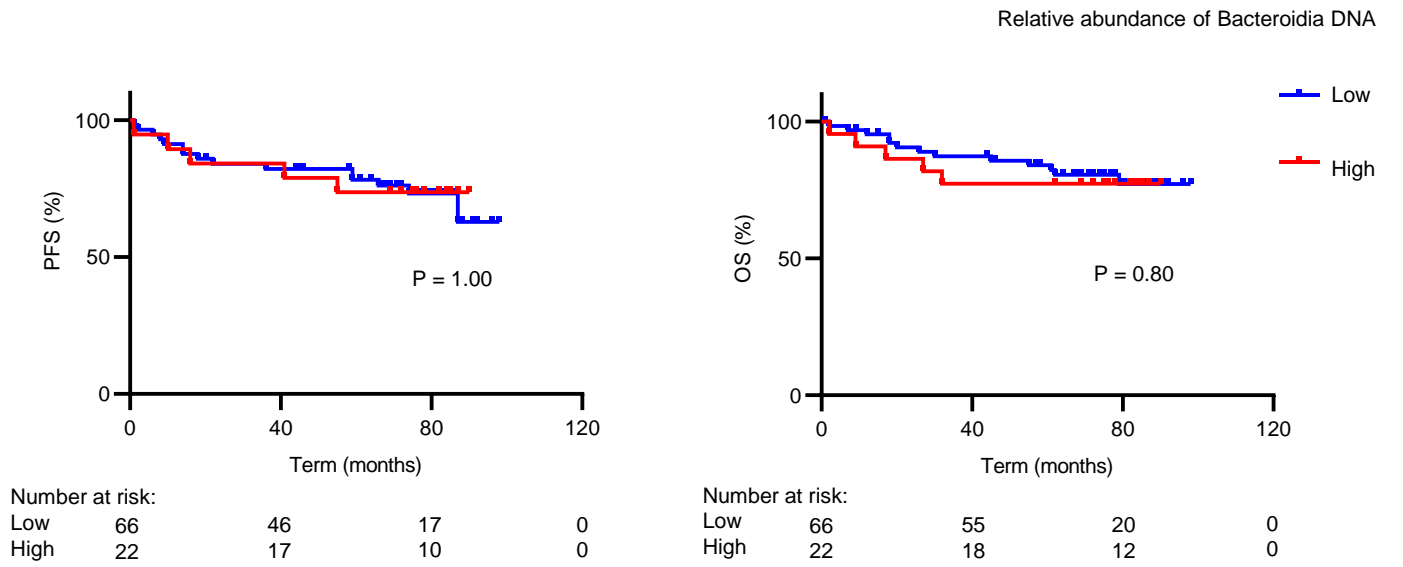

Supplement: Multimedia component 3 [file mmc3.pdf]

Figure S3

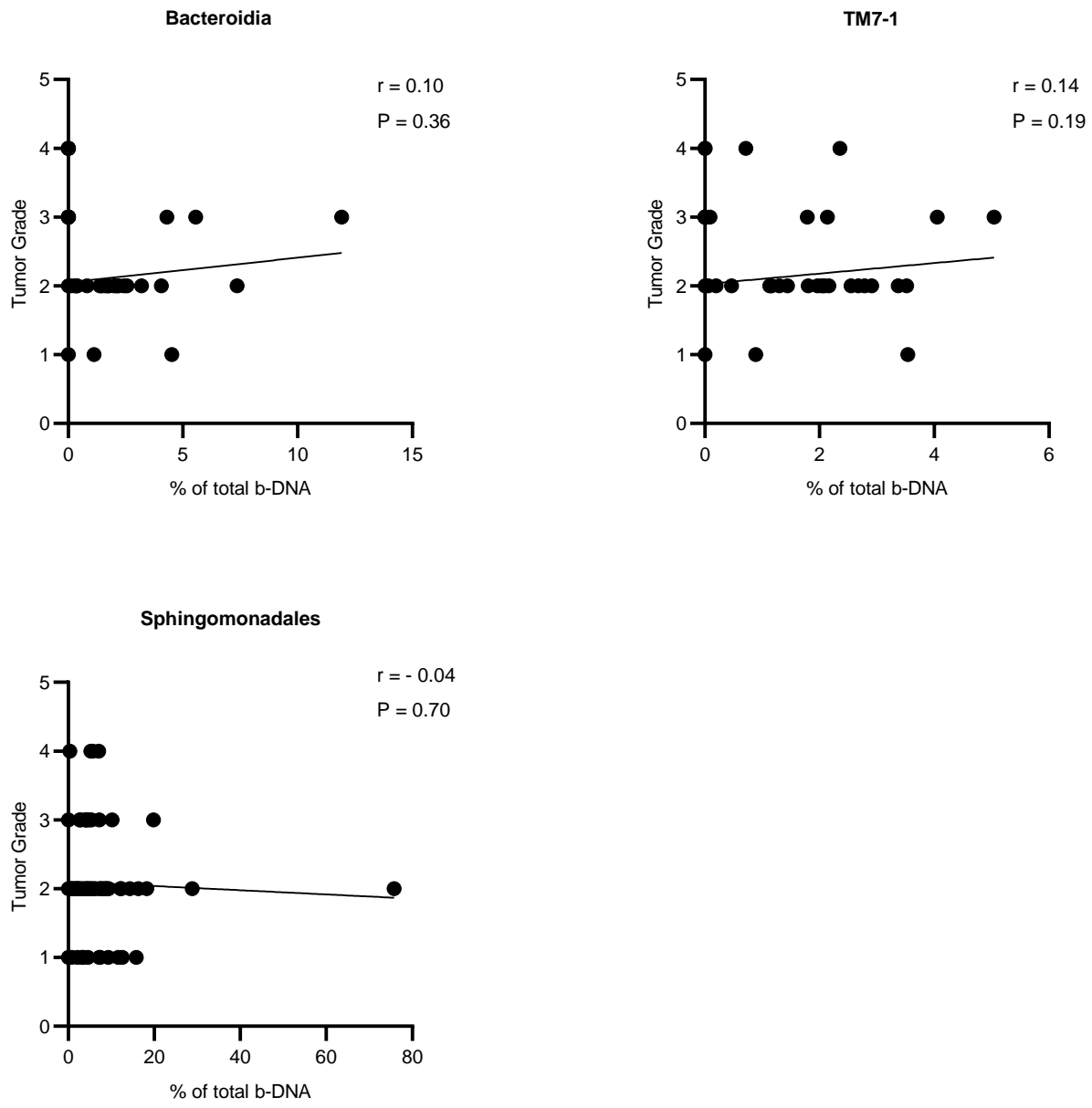

Supplement: Multimedia component 4 [file mmc4.pdf]
