# Supplementary material for: Bacteria-derived DNA in serum extracellular vesicles are biomarkers for renal cell carcinoma
Source: Heliyon. 2023 Sep 6;9(9):e19800. doi: 10.1016/j.heliyon.2023.e19800 (PMC10559165; doi:10.1016/j.heliyon.2023.e19800)
Supplement: Multimedia component 5 [file mmc5.docx]

**Table S1. PCR primer used in this study**

| **Target region** | **Forward (5’–3’)** | **Reverse (5’–3’)** |
| --- | --- | --- |
| **V1 – V2** | **27Fmod** | **338R** |
|  | (TCGTCGGCAGCGTCAGATGTGTATAAGAGACAGAGRGTTTGATYMTGGCTCAG) | (GTCTCGTGGGCTCGGAGATGTGTATAAGAGACAGTGCTGCCTCCCGTAGGAGT) |
