# Supplementary material for: Bacteria-derived DNA in serum extracellular vesicles are biomarkers for renal cell carcinoma
Source: Heliyon. 2023 Sep 6;9(9):e19800. doi: 10.1016/j.heliyon.2023.e19800 (PMC10559165; doi:10.1016/j.heliyon.2023.e19800)
Supplement: Multimedia component 6 [file mmc6.docx]

**Table S2. Patient’s characteristics in Cohort Z**

| Parameters |  | BC (n = 50) | HD (n = 20) | *P*-Value |
| --- | --- | --- | --- | --- |
| Age at operation, years | Median (Range) | 71 (47–90) | 62 (32–80) | **< 0.01** |
| BMI, kg/m^2^ | Median (Range) | 23.9 (17.8–37.6) | 21.7 (17.9–27.8) | 0.06 |
| Sex, n (%) | Male | 39 (78.0) | 5 (25.0) | **< 0.001** |
|  | Female | 11 (22.0) | 15 (75.0) |  |
| Histological type, n (%) | Urothelial carcinoma | 50 (100) |  |  |
| Pathological T stage, n (%) | Ta | 19 (38.0) |  |  |
|  | T1 | 15 (30.0) |  |  |
|  | T2 | 16 (32.0) |  |  |
| Clinical N stage, n (%) | N0 | 46 (92.0) |  |  |
|  | N1 - 3 | 4 (8.0) |  |  |
| Clinical M stage, n (%) | M0 | 48 (96.0) |  |  |
|  | M1 | 2 (4.0) |  |  |

Abbreviations: BC: bladder cancer; BMI: Body Mass Index; HD: healthy donor
