# Supplementary material for: Bacteria-derived DNA in serum extracellular vesicles are biomarkers for renal cell carcinoma
Source: Heliyon. 2023 Sep 6;9(9):e19800. doi: 10.1016/j.heliyon.2023.e19800 (PMC10559165; doi:10.1016/j.heliyon.2023.e19800)
Supplement: Multimedia component 7 [file mmc7.docx]

**Table S3. Correlation between TILs and b-DNA in serum EVs**

| Classification of CD4 T cells |  | Bacteroidia | | |  | TM7-1 | | |  | Sphingomonadales | | |
| --- | --- | --- | --- | --- | --- | --- | --- | --- | --- | --- | --- | --- |
|  |  | r | 95% CI | *P*-Value |  | r | 95% CI | *P*-Value |  | r | 95% CI | *P*-Value |
| Fr I (PD-1^-^ Tim-3^-^ CD4^+^) |  | -0.10 | -0.32–0.13 | 0.39 |  | 0.05 | -0.18–0.27 | 0.67 |  | -0.04 | -0.26–0.19 | 0.74 |
| Fr II (PD-1^low^ Tim-3^-^ CD4^+^) |  | 0.16 | -0.07–0.37 | 0.17 |  | 0.04 | -0.19–0.26 | 0.74 |  | -0.07 | -0.29–0.16 | 0.55 |
| Fr III (PD-1^high^ Tim-3^-^ CD4^+^) |  | -0.10 | -0.32–0.13 | 0.39 |  | -0.06 | -0.28–0.17 | 0.60 |  | 0.10 | -0.12–0.32 | 0.38 |
| Fr IV (PD-1^high^ Tim-3^+^ CD4^+^) |  | -0.05 | -0.27–0.18 | 0.70 |  | -0.16 | -0.37–0.07 | 0.17 |  | -0.04 | -0.26–0.18 | 0.72 |
| Fr V (PD-1^low^ Tim-3^+^ CD4^+^) |  | 0.40 | 0.19–0.57 | **< 0.001** |  | -0.04 | -0.26–0.18 | 0.72 |  | 0.01 | -0.21–0.24 | 0.91 |

Abbreviations: b-DNA: bacteria-derived DNA; TIL: tumor-infiltrating lymphocytes
